# Correlation between serum carbohydrate antigen 19-9 levels and computed tomography severity score in patients with nontuberculous mycobacterial pulmonary disease

**DOI:** 10.1038/s41598-021-82363-5

**Published:** 2021-02-02

**Authors:** Kangjoon Kim, Seung Hyun Yong, Su Hwan Lee, Sang Hoon Lee, Ah Young Leem, Song Yee Kim, Kyungsoo Chung, Eun Young Kim, Ji Ye Jung, Moo Suk Park, Young Sam Kim, Hye-Jeong Lee, Young Ae Kang

**Affiliations:** 1 Division of Pulmonary and Critical Care Medicine, Department of Internal Medicine, Severance Hospital, Yonsei University College of Medicine, Seoul, Republic of Korea; 2 Department of Radiology, Research Institute of Radiological Science, Severance Hospital, Yonsei University College of Medicine, Seoul, Republic of Korea; 3 Institute of Immunology and Immunological Disease, Yonsei University College of Medicine, Seoul, Republic of Korea

**Keywords:** Infectious diseases, Respiratory tract diseases, Predictive markers

## Abstract

There is no validated clinical biomarker for disease severity or treatment response for nontuberculous mycobacterial pulmonary disease (NTM-PD). We investigated the correlation between elevated serum carbohydrate antigen (CA) 19-9 levels and NTM-PD disease activity, defined using an imaging severity score based on chest computed tomography (CT). We retrospectively examined 79 patients with NTM-PD who underwent serum CA19-9 level assessments and chest CT less than 1 month apart. NTM-PD severity was rated using a CT-based scoring system. The correlation between the CT score and serum CA19-9 levels was evaluated. Chest CT revealed nodular bronchiectasis without cavitation in most patients (78.5%). Serum CA19-9 levels were elevated in 19 (24%) patients. Serum CA19-9 levels were positively correlated with the total CT score and bronchiectasis, bronchiolitis, cavity, and consolidation subscores. Partial correlation analysis revealed a significant positive correlation between serum CA19-9 levels and CT scores for total score and bronchiectasis, bronchiolitis, cavitation, and consolidation subscores after controlling for age, sex, and BMI. Serum CA19-9 levels were positively correlated with the CT severity score for NTM-PD. Serum CA19-9 may be useful in evaluating disease activity or therapeutic response in patients with NTM-PD.

## Introduction

The incidence of nontuberculous mycobacterial pulmonary disease (NTM-PD) and its associated disease burden have increased worldwide^1^. This is supposedly due to multiple etiologies including environmental^2^, bacterial, and host factors such as increased life expectancy^1^, a higher prevalence of immunocompromised conditions (e.g., immunosuppressive therapy, organ transplantation, chemotherapy)^3^, and an increase in the number of patients with comorbidities such as pre-existing lung disease, like chronic obstructive pulmonary disease^4^, cystic fibrosis^5^, or lung cancer^3,4^.

It is well-known that the diagnosis of NTM-PD itself does not always require initiation of treatment. Not all patients with NTM-PD progress to severe disease^6^, and the antimicrobial treatment of NTM-PD is frequently accompanied by adverse effects^7^. Therefore, it is essential to devise effective measures for the evaluation of NTM-PD disease activity to facilitate the assessment of disease progression and provide patients with timely and appropriate medical treatment.

The disease activity of NTM-PD is currently evaluated by the clinician, based on a comprehensive review of clinical symptoms, trends on chest imaging over time, or microbiologic investigations (e.g., sputum acid-fast bacillus smears and mycobacterial cultures)^8^. The decision to initiate treatment for NTM-PD is based on this assumption of disease activity or future possibility of disease progression, and potential adverse effects of treatment.

The following obstacles are to be considered while measuring disease activity: (1) the clinical signs are not disease-specific; (2) the time-trend pattern of chest imaging require months of follow-up and cannot reflect the disease activity of NTM-PD at a given point in time; (3) chest imaging is not always disease-specific, although some imaging features are known to be specific for NTM-PD^9^; and (4) microbiologic tests can evaluate the microbiological burden using smear positivity or culture positivity, but are relatively poor indicators with a crude scale, and the quality of the sputum specimen can affect test results^10^.

Consequently, there is dire need for a simple biomarker that reflects the disease activity of NTM-PD. Few studies have reported on the hematological biomarkers of NTM-PD, including IgA antibodies against glycopeptidolipids (GPL)^11^, serum Krebs von den Lungen-6 (KL-6)^12^, and carbohydrate antigen 19-9 (CA19-9)^13,14^.

We focused on a previous study, which suggested a correlation between active NTM-PD and serum CA19-9 levels^13^. In that study, patients with NTM-PD exhibited elevated serum CA19-9 levels, which plummeted significantly after treatment for NTM-PD. However, the previous study did not investigate the quantitative correlation between the disease activity and serum CA19-9 levels, which may be a useful biomarker.

Therefore, we aimed to further investigate the correlation between elevated serum CA19-9 levels and NTM-PD disease activity, which was defined using an imaging severity score based on chest computed tomography (CT).

## Methods

### Participants

This retrospective study examined patients with NTM-PD who visited Severance Hospital, a university-affiliated tertiary referral hospital in South Korea, between January 2005 and December 2019.

All procedures performed in studies involving human participants were in accordance with the ethical standards of the institutional and/or national research committee and with the 1964 Declaration of Helsinki and its later amendments or comparable ethical standards. The study was carried out under the permission of the Ethics Review Committee of Severance Hospital (IRB No. 4-2020-1103), and the need for informed consent was waived by the Committee because of the retrospective nature of this study.

**Figure 1:**
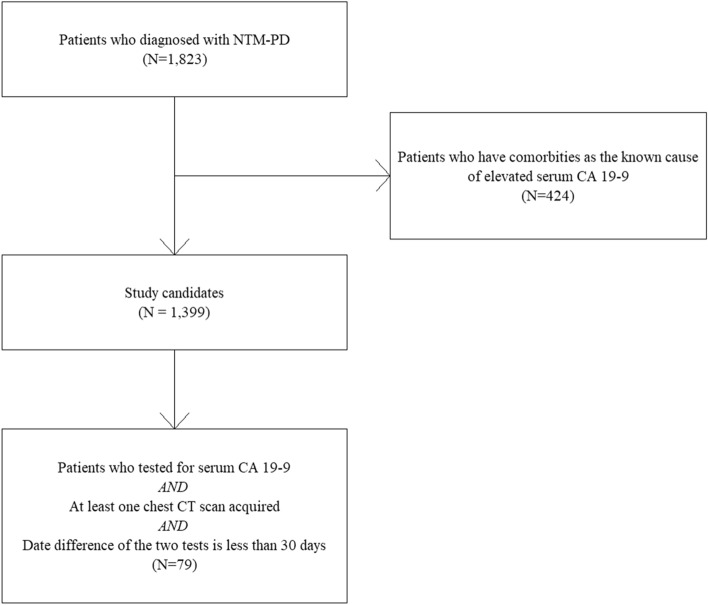
Selection process of the study population. *NTM-PD* nontuberculous mycobacterial pulmonary disease, *CA* carbohydrate antigen, *CT* computed tomography.

The diagnosis of NTM-PD was based on the American Thoracic Society/Infectious Diseases Society of America (ATS/IDSA) diagnostic criteria^15^. We excluded patients with comorbidities that could independently cause elevated serum CA19-9 levels from 1823 patients with NTM-PD. These comorbidities included stomach cancer^16^, colon cancer^17^, pancreatobiliary cancer^18,19^, lung cancer^20^, thyroid cancer^21^, benign hepatobiliary diseases^22,23^, thyroiditis^24^, and ovarian cysts^25,26^. The inclusion criteria were as follows: patients who underwent assessments for serum CA19-9 levels and chest CT scanning with an interval of less than 30 days between the two examinations and participants who underwent serum CA19-9 assessment in the time period between 1 month before the diagnosis and the initiation of NTM-PD treatment, which was considered as the active disease period of NTM-PD. We selected 79 patients for the final analysis, based on the above-mentioned eligibility criteria (Fig. 1). Because of the small number of participants, we additionally explored the data of patients with an extended time interval between chest CT acquisition and serum CA19-9 assessment up to 180 days, which accounted for an additional 17 participants. We performed supplementary analysis including those 17 additional participants.

### Data collection and measurement

We collected the patients’ demographic information, comorbidities, past medical history, symptom profile and progression of NTM, and treatment from their electronic medical records. The radiologic patterns of NTM-PD were categorized into the fibrocavitary (FC) type, nodular bronchiectatic (NB) type without cavitation, or NB type with cavitation. The NB form was characterized by bronchiectasis with multiple centrilobular nodules, and the FC form was defined by the presence of a FC lesion on chest CT^27^.

Serum CA19-9 levels were measured with the cobas 8000 modular analyzer (Roche Diagnostics GmbH, Mannheim, Germany) using the electrochemiluminescence immunoassay technique. The normal range was set at < 34 U/mL according to the manufacturer’s instructions.

### CT image evaluation

Chest CT images were obtained using 64-detector CT scanners (Discovery CT750 HD, GE Healthcare, Chicago, IL; Somatom Sensation 64, Siemens Healthineers, Erlangen, Germany) and a second-generation dual-source scanner (Somatom Definition Flash; Siemens Healthineers). According to the purpose of the CT scans (e.g., evaluating active hemoptysis or suspected pleural disease, workup for malignancy, ruling out pulmonary thromboembolism, etc.), iodinated contrast agents were used for 22 patients (27.8%, 22/79), and other patients (72.2%, 57/79) underwent non-contrast enhanced CT.

Iodinated contrast agents were used with an intravenous injection of 60 mL of contrast material (Iomeron 300, 400 mg I/mL; Bracco Imaging, Milan, Italy) into the antecubital vein at a flow rate of 4 mL/s following by a 20-mL saline chaser at a rate of 4 mL/s, triggering the scan from the main pulmonary artery. All scans were performed from the level of the supraclavicular fossae to the adrenal glands under an inspiratory breath-hold with following scanning parameters: tube voltage of 120 kVp, average tube current of 300 mA, average pitch of 0.9, and volume CT dose index less than 6.0 mGy. After scanning, axial images were reconstructed at a slice thickness of 1 mm and slice increment of 1 mm with a medium-smooth convolution kernel.

We adopted the following NTM-PD CT scoring system described by a previous study^28^ to evaluate the severity of NTM-PD. Bronchiectasis was considered to be present if the bronchial lumen diameter was greater than that of the adjacent pulmonary artery without tapering of the bronchial lumen diameter. Mucus plugging was considered to be present if a broad linear or branching attenuating lesion was observed within a proximal airway (lobar, segmental, or subsegmental bronchus) associated with airway dilatation.

Cellular bronchiolitis was defined as the presence of centrilobular small nodules (10 mm in diameter) and branching nodular structures (i.e., tree-in-bud sign) on the CT scans.

The presence of other abnormalities, including cavitations; nodules (10–30 mm in diameter); and lobular (consolidation measuring 10–20 mm in diameter with a polygonal shape), peribronchial, or segmental consolidation, was also recorded. A total CT score of 30 was allocated to determine the overall extent of lung lesions in each patient.

Two independent readers (one radiologist and one pulmonologist) evaluated the chest CT scans according to the scoring guidelines without being provided any clinical information about NTM-PD severity and CA19-9 levels.

### Statistical analysis

Descriptive variables were summarized using medians, interquartile ranges, and proportions. These variables were compared based on serum CA19-9 levels (normal or elevated) using the chi-squared test/Fisher’s exact test or the Mann–Whitney U test.

We calculated the interobserver agreement with respect to the extent and severity of bronchiectasis, cellular bronchiolitis, cavities, nodules, and consolidation using the intraclass correlation coefficient (ICC) because each observer evaluated the CT scans independently. The ICC was calculated based on a two-way random-effects model. The observer was allocated to the fixed-effect category, and the patient to the random-effect category. The ICC was considered to show poor agreement if its value was less than 0.5, moderate agreement if between 0.5 and 0.75, good agreement if between 0.75 and 0.9, and excellent agreement if greater than 0.90^29^.

The correlation between serum CA19-9 levels and the CT score for NTM-PD was analyzed using Spearman’s correlation coefficient and Spearman’s partial correlation coefficient, controlling for variables such as age, sex, and body mass index (BMI) for the latter test.

A *P*-value < 0.05 was considered significant for all analyses. Data analysis was performed using SPSS version 25 (IBM, Armonk, NY) and R (version 3.6.3; R Foundation for Statistical Computing, Vienna, Austria).

## Results

### Clinical characteristics

Of the 79 patients, 60 were women and 19 were men; the median age was 63 years (57–69 years). Twenty-three patients (29.1%) had a history of pulmonary tuberculosis and twelve patients (15.2%) had a history of diabetes mellitus. Expectoration of sputum (55.7%) and cough (44.3%) were the most common symptoms. *Mycobacterium avium complex* (MAC) was the most common pathogen (68.4%; *M. avium* 45.6%, *M. intracellulare* 22.8%, both 6.3%) and most patients (78.5%) presented with the NB type without cavitations on chest CT.

A clinician recommended antimicrobial treatment for NTM-PD in 37 of 79 (46.8%) patients during the follow-up period, and 6 patients refused treatment. Thus, only 31 (39.2%) patients received antimicrobial treatment. There was no significant difference in clinical characteristics such as sex, age, BMI, symptoms, NTM species, or treatment initiation between the groups of patients with and without elevated serum CA19-9. The proportion of patients with the FC type of NTM-PD seemed to be higher in the elevated CA19-9 group, but this was not significant (Table 1).

**Table 1:** Clinical characteristics of patients with pulmonary nontuberculous mycobacterial disease according to the serum CA19-9 level.

	Total	Normal CA19-9	Elevated CA19-9	*P*-value
	(N = 79)	(N = 60)	(N = 19)	
Sex, female	60 (75.9%)	45 (75.0%)	15 (78.9%)	0.494
Age, years	63 (56–68)	61 (55–68)	64 (60–72)	0.092
BMI	20.4 (18.4–22.7)	20.4 (18.4–21.9)	20.4 (17.4–23.0)	0.991
Smoker, current or former	16 (20.3%)	13 (21.7%)	3 (15.8%)	0.423
**Past history**				
Pulmonary TB	23 (29.1%)	19 (31.7%)	4 (21.1%)	0.550
HTN	21 (26.6%)	14 (23.3%)	7 (36.8%)	0.338
DM	12 (15.2%)	9 (15.0%)	3 (15.8%)	0.594
COPD	4 (5.1%)	2 (3.3%)	2 (10.5%)	0.243
CKD	3 (3.8%)	2 (3.3%)	1 (5.3%)	0.567
**Symptom**				
Cough	35 (44.3%)	26 (43.3%)	9 (47.4%)	0.965
Sputum	44 (55.7%)	34 (56.7%)	10 (52.6%)	0.965
Dyspnea	11 (14.9%)	9 (15.0%)	2 (10.5%)	0.476
Hemoptysis	18 (22.8%)	11 (18.3%)	7 (36.8%)	0.089
Fever	3 (3.8%)	3 (5.0%)	0 (0.0%)	0.433
Weakness	4 (5.1%)	3 (5.0%)	1 (5.3%)	0.675
Weight loss	5 (6.3%)	4 (6.7%)	1 (5.3%)	0.653
**Species**				0.480
*MAC*	54 (68.4%)	39 (65.0%)	15 (78.9%)	
*M. abscessus*	17 (21.5%)	13 (25.0%)	2 (10.5%)	
*MAC* + *M. abscessus*	6 (7.6%)	6 (6.7%)	2 (10.5%)	
Others	2 (2.5%)	2 (3.3%)	0 (0.0%)	
Treatment recommendation	37 (46.8%)	25 (41.7%)	12 (63.2%)	0.120
**Radiologic type**				0.077
FC type	4 (5.1%)	1 (1.7%)	3 (15.8%)	
NB type with cavitation	13 (16.5%)	10 (16.7%)	3 (15.8%)	
NB type without cavitation	62 (78.5%)	49 (81.7%)	13 (68.4%)	
CA 19-9 (U/mL)	15.1 (9.2–33.7)	12.9 (6.5–16.3)	67.6 (54.5–108.0)	0.000

Values are expressed as numbers (%) or medians (1Q–3Q).

*CA* carbohydrate antigen, *BMI* body mass index, *HTN* hypertension, *DM* diabetes mellitus, *COPD* chronic obstructive pulmonary disease, *CKD* chronic kidney disease, *MAC Mycobacterium avium complex*, *FC* fibrocavitary, *NB* nodular bronchiectatic.

### Interobserver agreement

We calculated the ICC for each subcategory of the CT score and the total score to evaluate the interobserver agreement for CT scoring for NTM-PD severity.

The ICC for the total score exhibited good agreement (0.87, 95% CI 0.76–0.93). The ICC for the “cavitation” score showed good agreement (0.81, 95% CI 0.72–0.88), those for “bronchiectasis” and “cellular bronchiolitis” showed moderate agreement (ICC 0.73, 0.51, respectively), and those for “nodules” and “consolidation” showed poor agreement (ICC 0.26 and 0.27, respectively) (Table 2).

**Table 2:** ICC for each pattern of lung abnormality.

Pattern	ICC	95% CI
Bronchiectasis	0.73	0.60–0.82
Cellular bronchiolitis	0.51	0.16–0.71
Cavitation	0.81	0.72–0.88
Nodules	0.26	0.04–0.45
Consolidation	0.27	0.02–0.48
Total CT score	0.87	0.76–0.93

*ICC* intraclass correlation, *CI* confidence interval, *CT* computed tomography.

### Correlation between NTM-PD CT score and serum CA19-9 level

We divided and compared the participants according to their serum CA19-9 levels (normal or elevated). Those with elevated serum CA19-9 had significantly higher total CT scores and bronchiectasis and cavitation subscores (P < 0.05) (Table 3).

**Table 3:** CT scores of patients with pulmonary nontuberculous mycobacterial disease, according to the serum CA19-9 level.

Category	Normal CA19-9 (N = 60)	Elevated CA19-9 (N = 19)	*P*-value
Total (30 points)	7.8 (6.0–10.5)	12.5 (9.5–14.0)	0.001
Bronchiectasis (9 points)	3.0 (2.0–4.0)	4.0 (3.0–4.5)	0.031
Cellular bronchiolitis (6 points)	2.5 (2.0–4.0)	3.5 (2.5–5.0)	0.099
Cavitation (9 points)	0.0 (0.0–0.0)	2.5 (0.0–4.0)	0.021
Nodules (3 points)	0.5 (0.5–1.5)	0.5 (0.5–1.0)	0.318
Consolidation (3 points)	1.0 (0.5–1.5)	1.5 (1.0–1.5)	0.096

Values are presented as medians (Q1–Q3).

*CT* computed tomography, *CA* carbohydrate antigen.

**Figure 2:**
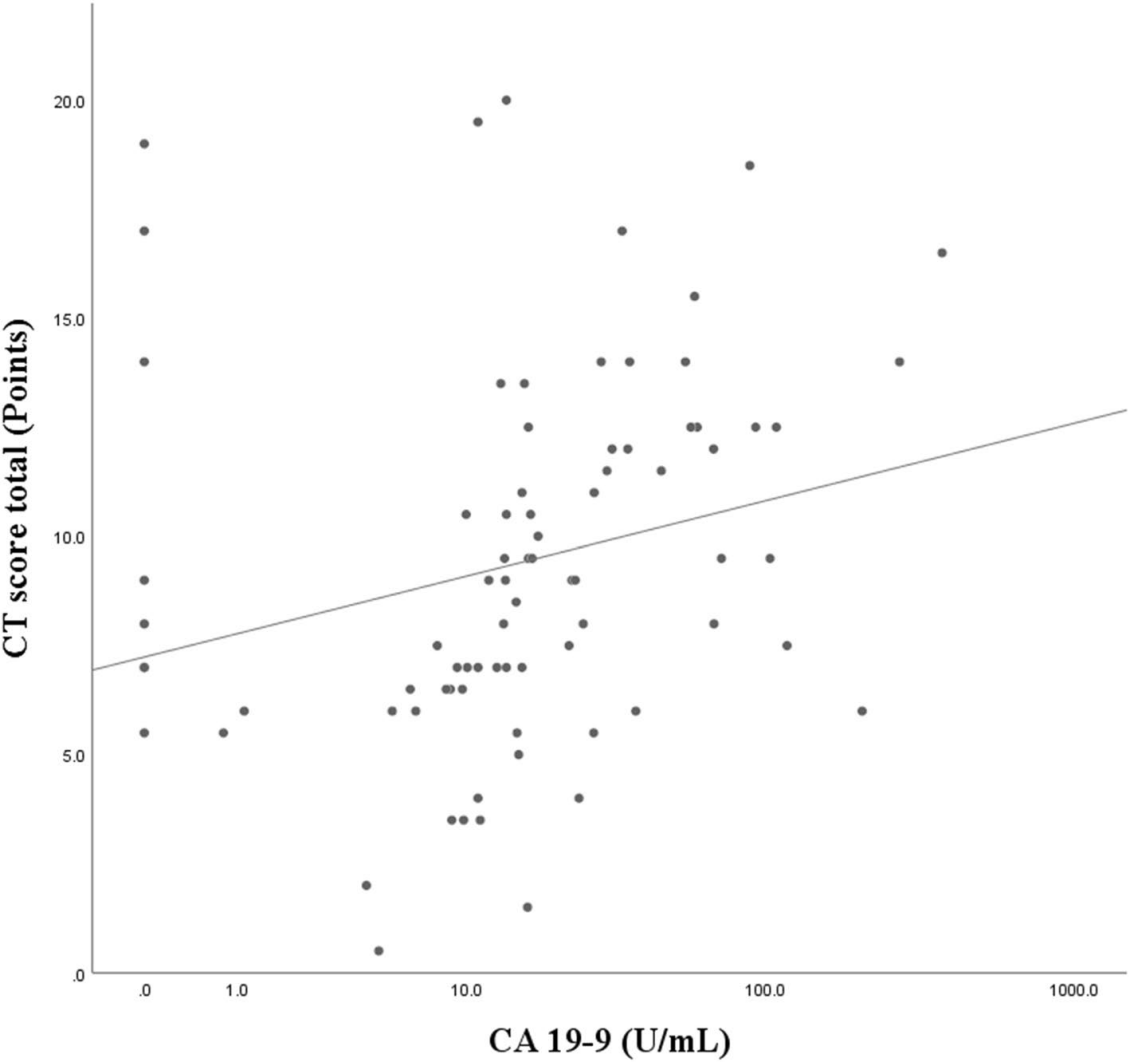
Scatter plot depicting the total CT score total and serum CA19-9 levels. *CT* computed tomography, *CA* carbohydrate antigen.

Correlation analysis revealed significant positive correlations between serum CA19-9 levels and NTM-PD CT scores (total score and bronchiectasis, bronchiolitis, cavity, and consolidation subscores) and a negative correlation between the total score and BMI (Table 4, Fig. 2). Age and BMI were not correlated with the serum CA19-9 level (Table 4).

**Table 4:** Result of correlation analysis using Spearman’s correlation coefficient.

	Age	BMI	Bronchiectasis	Bronchiolitis	Cavitation	Nodules	Consolidation	Total	Serum CA19-9
Age	1								
BMI	− 0.080	1							
Bronchiectasis^a^	0.244*	− 0.374**	1						
Bronchiolitis^a^	0.090	− 0.116	0.301**	1					
Cavitation^a^	0.034	− 0.255*	0.320**	0.175	1				
Nodules^a^	0.068	− 0.171	0.059	0.151	− 0.001	1			
Consolidation^a^	0.284*	− 0.275*	0.322**	0.431**	0.331**	0.072	1		
Total^a^	0.154	− 0.358**	0.691**	0.630**	0.737**	0.191	0.618**	1	
Serum CA19-9	0.171	− 0.043	0.251*	0.289**	0.221*	0.137	0.303**	0.416**	1

*BMI* body mass index, *CA* carbohydrate antigen.

*Level of significance is 0.05, **Level of significance is 0.01.

^a^Categories of the computed tomography scoring system.

Partial correlation analysis revealed a significant positive correlation between the serum CA19-9 level and NTM-PD CT subscores for bronchiectasis, bronchiolitis, cavity, consolidation, and the total score, after controlling for age, sex, and BMI (Table 5).

**Table 5:** Partial correlation coefficient for CA19-9 and CT score for NTM-PD.

Category	Spearman’s partial r (95% CI)^a^
Bronchiectasis	0.2512 (0.1152–0.3793)
Bronchiolitis	0.2907 (0.1509–0.4040)
Cavitation	0.1567 (0.0004–0.3885)
Nodule	0.1450 (− 0.0155 to 0.2785)
Consolidation	0.3039 (0.1671–0.4122)
Total score	0.4162 (0.2846–0.5186)

*CA* carbohydrate antigen, *CT* computed tomography, *NTM-PD* nontuberculous mycobacterial pulmonary disease, *CI* confidence interval, *BMI* body mass index.

^a^The controlled variables included age, sex and BMI.

In the supplementary analysis including additional 17 participants, the positive correlation between serum CA19-9 levels and NTM-PD CT score was still statistically significant (total score and bronchiolitis, cavity, and consolidation subscores; Table S2, Figure S1), and partial correlation analysis also revealed a significant positive correlation between serum CA19-9 levels and NTM-PD CT scores (total score and bronchiectasis, bronchiolitis, cavity, and consolidation subscores) after controlling for age, sex, and BMI (Table S3).

## Discussion

This study sought to conduct a detailed investigation on the correlation between an increase in serum CA19-9 levels and NTM-PD disease activity, which was defined by the CT severity score.

In this study, the serum CA19-9 level was significantly correlated with the chest CT severity score in patients with NTM-PD after adjusting for age, sex, and BMI. Considering the wide range of serum CA19-9 levels (within normal limits) in patients with NTM-PD in this study, its utility as a diagnostic marker for NTM-PD was apparently limited; however, it may be useful in assessing the severity of NTM-PD.

CA19-9 is the most commonly used serum marker for pancreatic cancer. CA19-9, which is synthesized by normal human pancreatic and biliary ductal cells and gastric, colonic, endometrial, and salivary epithelia, is present in small amounts in serum, and may be elevated in several benign or malignant gastrointestinal disorders^30^.

Moreover, increased CA19-9 levels have been observed in patients with several pulmonary diseases^31^. Studies have shown that patients with idiopathic interstitial pneumonia (IIP)^32^, collagen disease-associated pulmonary fibrosis^33^, diffuse panbronchiolitis (DPB)^34^, and bronchiectasis^31^ had elevated serum CA19-9 levels.

The mechanism responsible for the elevation of serum CA19-9 in pulmonary diseases has not fully elucidated. However, previous studies using immunohistochemical staining reported that CA19-9 was selectively expressed in bronchiolar epithelial cells in patients with IIP and DPB, whereas alveolar epithelial cells or bronchiolar glandular cells were negative for CA19-9, irrespective of the etiology^32,34^. Moreover, there was good correlation between the degree of inflammation and antigen expression and between the intensity of staining and antigen levels in bronchoalveolar lavage fluid^34^. KL-6, which has been previously shown to be a potential biomarker of NTM-PD^12^, was also shown to be strongly expressed in bronchiolar epithelial cells^35^. These findings indicate that inflammation and damage to bronchiolar epithelial cells may be responsible for the elevation in serum CA19-9 in chronic airway inflammatory diseases.

CT imaging indicates that small and large airways are the major sites of involvement in NTM-PD, which manifest with inflammatory bronchiolitis and bronchiectasis. Histopathologically, granulomatous inflammation of the airway walls is correlated with imaging findings^36^. Although the assessment of the disease-specific activity and severity of NTM-PD is one of the most important factors in treating patients with NTM-PD, none of the known assessment tools are appropriate for NTM-PD. From the various clinical, radiological, and microbiological assessments, radiographic examinations have played an essential role in the diagnosis of NTM-PD and also in determining the extent of the disease and assessing the response to treatment. CT imaging has considerably improved the assessment of this disease. Several scoring criteria have been devised for grading the disease severity of NTM-PD based on CT imaging. In the present study, the CT scores of the severity of lung involvement in NTM-PD were calculated using a modified version of a previously published scoring system proposed by Song et al.^37^ A previous study found that the scoring system was correlated with the results of the pulmonary function test and functional impairment in patients with MAC pulmonary disease and is also used to assess the treatment response of *M. abscessus* and *M. massiliense* pulmonary disease^28^.

The prognostic value and effect of the extent of the disease or number of lung segments affected by NTM-PD on chest CT on the health-related quality of life of patients with NTM-PD have been investigated^38–41^. Thus, the good correlation between serum CA19-9 levels and CT scores of patients with NTM-PD in our study suggests that serum CA19-9 levels may be used as a biomarker of the disease severity of NTM-PD.

Blood monitoring-based biomarkers, correlated with NTM-PD disease activity, may provide clinicians with reliable clues regarding the appropriate time to initiate treatment or information of treatment response during the course of treatment. Although some researchers have proposed a few biomarkers (i.e., anti-GPL IgA^11^, type I cytokine-associated molecules^42^, and KL-6^12^) for assessing the response to therapy, they have not been validated sufficiently to be used in daily practice.

Hong et al.^13^ examined the clinical significance of CA19-9 as a biomarker for monitoring treatment for NTM-PD. In that study, patients with good treatment response showed a significant decline in the CA19-9 level, while no difference was observed in the CA19-9 level between before and after treatment in non-responders.

Our study sought to reveal a direct correlation between serum CA19-9 and NTM-PD disease activity, which was reflected by the severity score based on chest CT imaging. We found a positive correlation between serum CA19-9 levels and the CT severity score for NTM-PD, which means that serum CA19-9 could be a potential biomarker for NTM-PD. Further studies are needed, especially those focusing on serial trends in serum CA19-9 levels following disease progression or antimicrobial treatment.

Our study had a few limitations. First, we could not exclude patients with a negative Lewis antigen phenotype, because the Lewis antigen test was not performed routinely for all patients owing to the retrospective nature of the cohort study. Serum levels of CA19-9 are not elevated in patients with the Lewis negative phenotype^43^. Therefore, there is a possibility of false-negative findings with respect to the serum CA19-9 levels in our study population, which means that the actual magnitude of the positive correlation may be greater. A study that evaluated blood samples from 225 healthy Korean volunteers found that 11.1% of participants exhibited the Lewis negative phenotype^44^. Second, our study included a small sample with a retrospective study design, not including a control group. A group of participants with bronchiectasis without NTM-PD might be a good control. Third, we could not evaluate the effect of treatment, because serial measurements of serum CA19-9 levels were not available.

In conclusion, serum CA19-9 levels showed a positive correlation with the CT severity score for NTM-PD, especially the total score and subscores for bronchiectasis, bronchiolitis, cavitation, and consolidation. Serum CA19-9 may be a useful marker in evaluating disease activity or therapeutic response in patients with NTM-PD, and further studies on this topic are warranted.

## Supplementary Information

Supplementary Information.

## Data Availability

The data that support the findings of this study are available from the corresponding author, upon reasonable request.
