# Supplementary material for: Correlation between serum carbohydrate antigen 19-9 levels and computed tomography severity score in patients with nontuberculous mycobacterial pulmonary disease
Source: Sci Rep. 2021 Feb 2;11:2777. doi: 10.1038/s41598-021-82363-5 (PMC7854612; doi:10.1038/s41598-021-82363-5)
Supplement: Supplementary file 1 — Supplementary Information. [file 41598_2021_82363_MOESM1_ESM.docx]

Correlation between serum carbohydrate antigen 19-9 levels and computed tomography severity score in patients with nontuberculous mycobacterial pulmonary disease

Kangjoon Kim^1^, Seung Hyun Yong^1^, Su Hwan Lee^1^, Sang Hoon Lee^1^, Ah Young Leem^1^, Song Yee Kim^1^, Kyungsoo Chung^1^, Eun Young Kim^1^, Ji Ye Jung^1^, Moo Suk Park^1^, Young Sam Kim^1^, Hye-Jeong Lee^2^*, Young Ae Kang^1,3^*

^1^ Division of Pulmonary and Critical Care Medicine, Department of Internal Medicine, Severance Hospital, Yonsei University College of Medicine, Seoul, Republic of Korea

^2^ Department of Radiology, Severance Hospital, Yonsei University College of Medicine, Seoul, Republic of Korea

^3^ Institute of Immunology and Immunological Disease, Yonsei University College of Medicine, Seoul, Republic of Korea

* Co-corresponding author

* E-mail address: [mdkang@yuhs.ac](mailto:mdkang@yuhs.ac) (Y. Kang), [IAMOTWO@yuhs.ac](mailto:IAMOTWO@yuhs.ac) (H. Lee)

___________________________________________________________________________

Figure S1. Scatter plot of 96 participants within the expanded inclusion criteria (i.e., the date difference of CT acquisition and measurement of serum CA19-9 level is 180 or less), depicting the total CT score total and serum CA19-9 levels


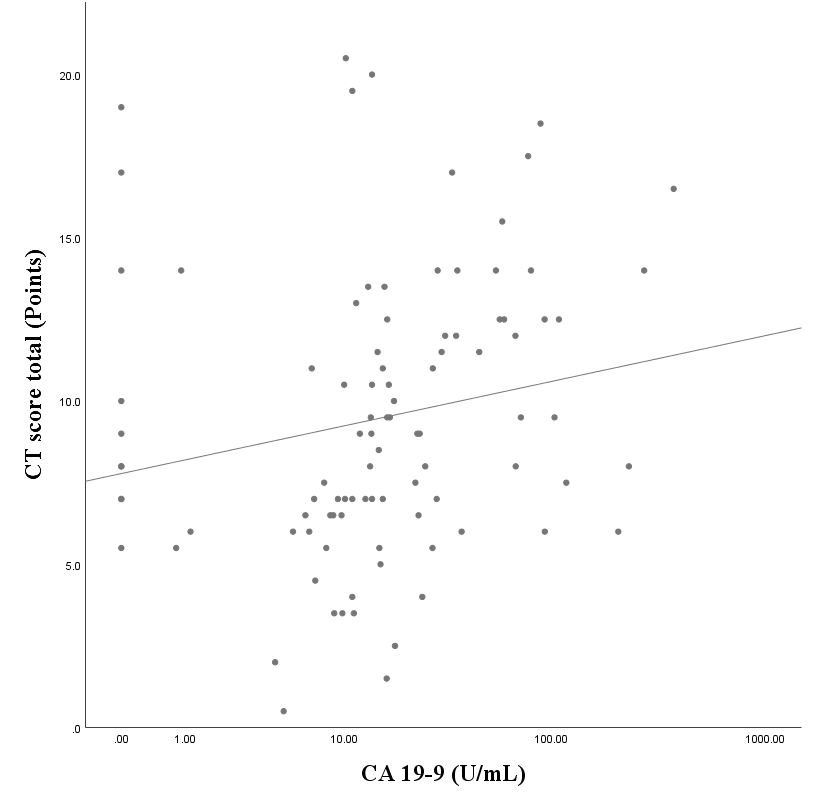


Table S1. CT scores of 96 participants within the expanded inclusion criteria, according to the serum CA19-9 level

| Category | Normal  CA19-9  (N=73) | Elevated  CA19-9  (N=23) | *P*-value |
| --- | --- | --- | --- |
| Total (30 points) | 8.0 (6.0-11.0) | 12.5 (8.0-14.0) | 0.001 |
| Bronchiectasis (9 points) | 3.0 (2.0-4.0) | 3.5 (2.5-5.0) | 0.068 |
| Cellular bronchiolitis (6 points) | 2.5 (2.0-4.0) | 3.5 (2.5-5.0) | 0.068 |
| Cavitation (9 points) | 0.0 (0.0-0.0) | 2.5 (0.0-4.0) | 0.008 |
| Nodules (3 points) | 0.5 (0.0-1.0) | 0.5 (0.5-1.0) | 0.377 |
| Consolidation (3 points) | 1.0 (0.5-1.5) | 1.5 (1.0-1.5) | 0.056 |

Values are presented as medians (Q1–Q3).

CT = computed tomography; CA = carbohydrate antigen

Table S2. Correlation analysis of 96 patients within the expanded inclusion criteria, using Spearman’s correlation coefficient

|  | Age | BMI | Bronchiectasis | Bronchiolitis | Cavitation | Nodules | Consolidation | Total | Serum CA19-9 |
| --- | --- | --- | --- | --- | --- | --- | --- | --- | --- |
| Age | `1 |  |  |  |  |  |  |  |  |
| BMI | -.108 | `1 |  |  |  |  |  |  |  |
| Bronchiectasis† | `.154 | -.367^**^ | `1 |  |  |  |  |  |  |
| Bronchiolitis† | `.002 | -.066 | `.347^**^ | `1 |  |  |  |  |  |
| Cavitation† | -.042 | -.197 | `.332^**^ | `.178 | `1 |  |  |  |  |
| Nodules† | `.021 | -.119 | `.037 | `.165 | `.031 | `1 |  |  |  |
| Consolidation† | `.189 | -.217^*^ | `.358^**^ | `.440^**^ | `.345^**^ | `.057 | `1 |  |  |
| Total† | `.050 | -.302^**^ | `.721^**^ | `.642^**^ | `.725^**^ | `.177 | `.642^**^ | `1 |  |
| Serum CA19-9 | `.152 | `.028 | `.135 | `.247^*^ | `.206^*^ | `.077 | `.249^*^ | `.318^**^ | `1 |

† Categories of the computed tomography scoring system

^*^ Level of significance is 0.05 ^**^ Level of significance is 0.01

BMI = body mass index, CA = carbohydrate antigen

Table S3. Partial correlation coefficient for CA19-9 and CT score for NTM-PD, from 96 participants within the expanded inclusion criteria

| Category | Spearman’s partial r (95% CI) * | |
| --- | --- | --- |
| Bronchiectasis | 0.131 | ( 0.020 - 0.262) |
| Bronchiolitis | 0.242 | ( 0.134 - 0.352) |
| Cavitation | 0.149 | ( 0.002 - 0.354) |
| Nodule | 0.067 | (-0.048 - 0.210) |
| Consolidation | 0.249 | ( 0.128 - 0.352) |
| Total score | 0.317 | ( 0.208 - 0.420) |

*The controlled variables included age, sex and BMI.

CA = carbohydrate antigen; CT = computed tomography; NTM-PD = nontuberculous mycobacterial pulmonary disease; CI = confidence interval; BMI = body mass index
